# Coronary Artery Occlusion with Sharp Blood Pressure Drop during General Anesthesia Induction: A Case Report

**DOI:** 10.3390/medicina60020232

**Published:** 2024-01-29

**Authors:** Jae Young Ji, Yong Han Seo, Ho Soon Jung, Hea Rim Chun, Jin Soo Park, Woo Jong Kim, Jae Min Ahn, Yu Jun Park, Ye Eun Shin, Chan Ho Park

**Affiliations:** 1 Department of Anesthesiology and Pain Medicine, Soonchunhyang University Hospital Cheonan, 31, Suncheonhyang 6-gil, Dongam-gu, Cheonan 31151, Republic of Korea; 98303@schmc.ac.kr (J.Y.J.); dyflam@schmc.ac.kr (H.S.J.); blau00@schmc.ac.kr (H.R.C.); 118541@schmc.ac.kr (J.S.P.); 135709@schmc.ac.kr (Y.J.P.); sye0822@gmail.com (Y.E.S.); 2 Department of Orthopaedic Surgery, Soonchunhyang University Hospital Cheonan, 31, Suncheonhyang 6-gil, Dongam-gu, Cheonan 31151, Republic of Korea; kwj9383@hanmail.net; 3 Department of Neurosurgery, Soonchunhyang University Hospital Cheonan, 31, Suncheonhyang 6-gil, Dongam-gu, Cheonan 31151, Republic of Korea; jmstarry21@naver.com; 4 Department of Radiology, Soonchunhyang University Hospital Cheonan, 31, Sooncheonhyang 6-gil, Donam-gu, Cheonan 31151, Republic of Korea; 98480@schmc.ac.kr

**Keywords:** coronary artery disease, dehydration, propofol, sevoflurane

## Abstract

Most anesthetics reduce cardiac functions and lower blood pressure (BP), potentially causing excessive BP reduction in dehydrated patients or those with heart conditions, such as coronary artery disease (CAD). Considering the increased prevalence of cardiovascular disease with age, anesthesiologists must be cautious about BP reduction during general anesthesia in older adults. In the present case, a 76-year-old male patient with undiagnosed CAD in a hypovolemic state experienced a significant drop in systolic BP to the fifties during propofol and sevoflurane anesthesia. Despite the use of vasopressors, excessive hypotension persisted, leading to anesthesia suspension. Subsequent cardiac examinations, including computed tomography heart angio and calcium score, and coronary angiogram, revealed a near total occlusion of the proximal left anterior descending coronary artery (pLAD) and the formation of collateral circulation. After 5 days of hydration and anticoagulation medications and confirmation of normovolemic state, general anesthesia was attempted again and successfully induced; a normal BP was maintained throughout the surgery. Thus, it is important to conduct a thorough cardiac evaluation and maintain normovolemia for general anesthesia in older adults.

## 1. Introduction

Approximately 15% of patients who undergo non-cardiac surgery under general anesthesia develop cardiac-related complications [1]. A study in the United States demonstrated that the prevalence of cardiac diseases increases with age, reaching 75% among individuals aged 60–79 years and 86% among those aged ≥80 years. Furthermore, cardiac complications were reported as major a factor for consideration when administering anesthesia to older adults [2]. In a dehydrated state, the compensatory cardiovascular system to maintain normal blood pressure (BP) is disturbed by most anesthetic agents and can lead to severe hypotension during anesthesia [3]. Moreover, in patients with coronary artery disease (CAD), there is a higher risk of developing fatal complications that can lead to cardiac arrest if hypotension occurs following the administration of anesthetics, such as sevoflurane or propofol; this is attributable to the resulting impaired blood flow to the heart [4]. We present a case of an undiagnosed coronary artery occlusion and suspected dehydration in which severe hypotension developed immediately after the induction of general anesthesia for total hip replacement arthroplasty, leading to immediate anesthesia discontinuation. Herein, we discuss the relationship between general anesthesia, intravascular volume, and heart diseases by reviewing this case.

## 2. Case Presentation

**Figure 1:**
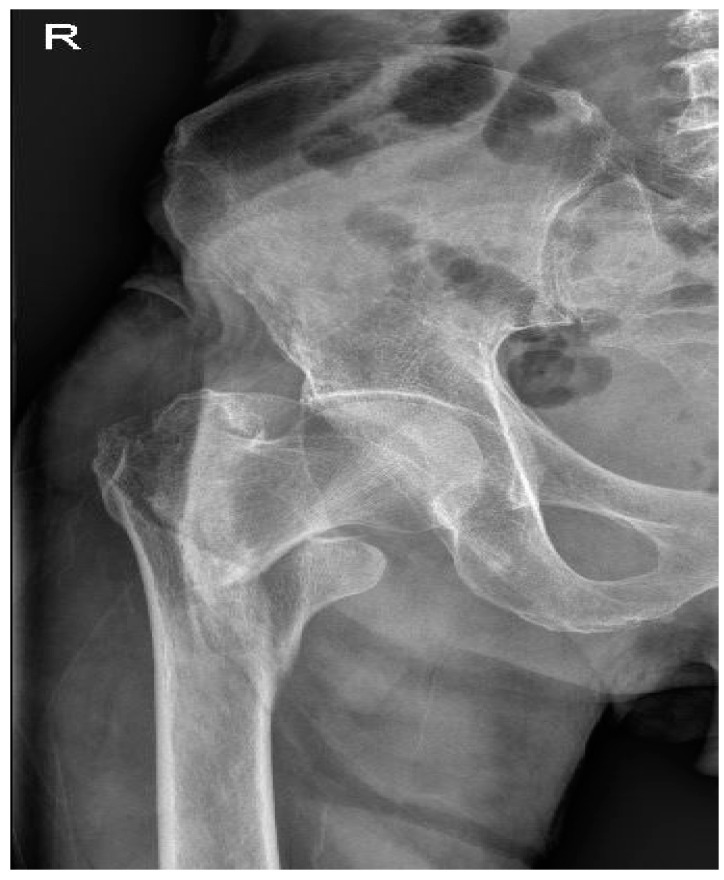
Fracture of the right hip femur intertrochanter (Pelvis AP (Anteroposterior) X-ray).

We obtained prior informed consent from the patient and approval from Institutional Review Board and Human Research Ethics Committee of Soonchunhyang University Cheonan Hospital (approval no.: 2023-10-034; date of approval: 3 November 2023) to publish this case report. The patient was a 76-year-old male with a history of hypertension and type 2 diabetes mellitus (DM). Prior to the surgery, the patient had been taking valsartan (20 mg), aspirin (100 mg), lasix (20 mg), and linagliptin (5 mg) daily for hypertension and diabetes mellitus, as well as two subcutaneous injections of ultra-long-acting insulin per day. He was diagnosed with a right hip femur intertrochanter fracture (Figure 1) and was scheduled for total hip replacement arthroplasty under general anesthesia. Echocardiography performed 3 years prior showed a left ventricular ejection fraction (LVEF) of 68%, and a second echocardiography was not performed until the anesthesia.

**Figure 2:**
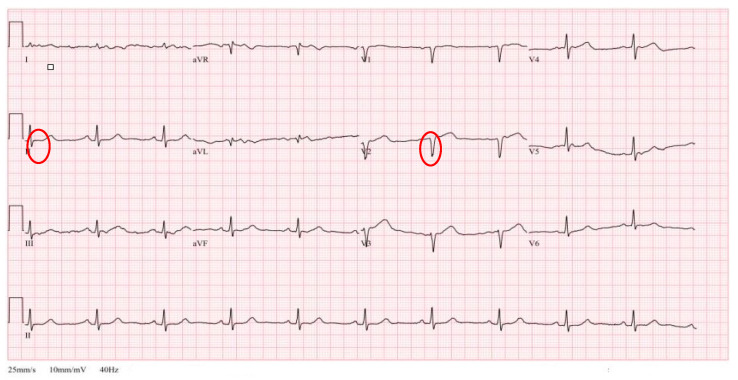
Pre-anesthesia EKG showed no remarkable findings regarding the ST segment on Lead II (Circle) and Showed pathologic Q wave on V2 (Circle).

An electrocardiogram (EKG) performed immediately before the anesthesia (Figure 2) showed pathologic Q waves on V2, based on which septal infraction was suspected. However, since myocardial infarction could not be diagnosed solely based on abnormal EKG findings [5] and the patient maintained a BP of 150/70 mmHg before induction of anesthesia, we decided to proceed with the surgery without additional cardiac evaluation so that the patient can ambulate as soon as possible, considering his old age. Prior to inducing anesthesia, EKG, non-invasive blood pressure, pulse oximeter, and bispectral index monitoring devices were placed on the patient. Due to a pleth variability index (PVI) of 16% shown on the pulse oximeter before induction, we suspected dehydration. However, the patient had higher-than-normal BP, was communicating well, and did not complain of symptoms such as poor physical state, and propofol—a commonly used anesthetic—was used for the induction. As the required propofol dosage for induction decreases with advancing age [6], 1 mg/kg of propofol was administered. After loss of consciousness, sevoflurane 1 MAC was administered to maintain the anesthesia. Subsequently, rocuronium (0.8 mg/kg) was administered for muscle block. After the induction of anesthesia, BP dropped to 54/38 mmHg; therefore, ephedrine 8 mg (vasopressor) was administered. Subsequently, BP increased to 83/54 mmHg and endotracheal intubation was performed. Due to the airway stimulation from intubation, BP was elevated to 134/78 mmHg. Following radial arterial cannulation, stroke volume variation (SVV) measured on Vigileo FloTrac system©WT McGee MD 2005 was 16%, again confirming hypovolemia. Further, the cardiac index was 2.1 L/min/m^2^, suggesting impaired cardiac functions. A few minutes later, BP dropped again to 58/32 mmHg, and although a bolus of epinephrine (30 mcg) temporarily elevated the BP to 120/60 mmHg, the systolic blood pressure (SBP) again dropped to the 60 s. In addition, there was a clear new-onset ST depression observed on the EKG after induction, suggestive of sudden cardiac ischemia. As total hip arthroplasty may involve massive bleeding, normal BP was not maintained after the administration of anesthetics, and EKG showed ischemic findings, the anesthesiologist determined that the anesthesia should be discontinued. Upon discussion with the surgeon, anesthesia was discontinued. Subsequently, sevoflurane was stopped, and the patient’s vital signs measured immediately after the patient woke up were as follows: BP, 93/55 mmHg; HR, 71 beats/min. The patient was transferred to the intensive care unit from the operating room, and the initial BP assessment showed that the BP had recovered to the pre-anesthesia state (143/88 mmHg). Emergent echocardiography performed after anesthesia showed mild left ventricular dysfunction and near-normal ejection fraction at 45–50%.

**Figure 3:**
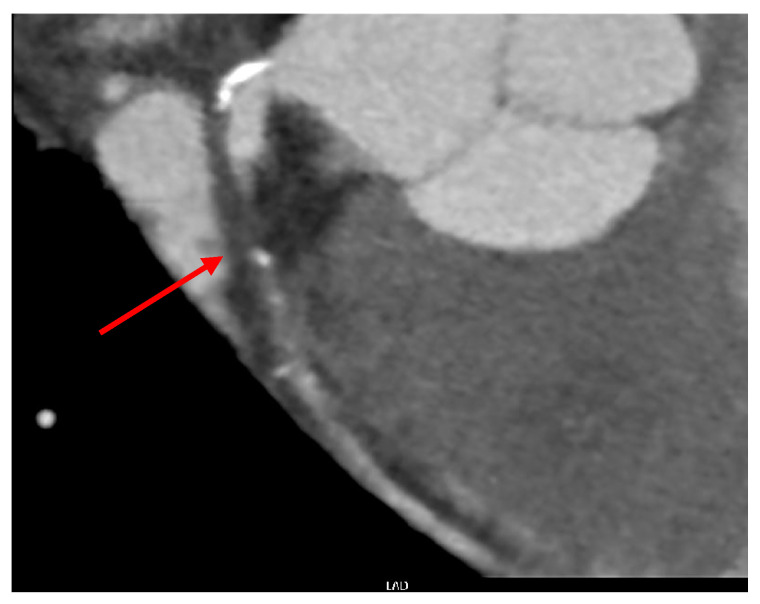
Near total occlusion of the left main descending artery (Arrow) indicated on CT heart angio and calcium score.

Computed tomography (CT) heart angio and calcium score were performed, as recommended per cardiologist consult, and near total occlusion of the proximal left anterior descending artery (pLAD) was found (Figure 3).

**Figure 4:**
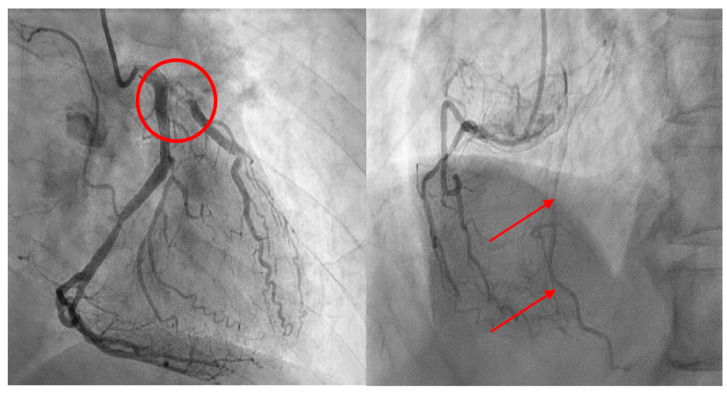
Coronary angiogram shows occlusion of the Proximal left anterior descending artery (Circle) (**Left**) and formation of collateral circulation in the right artery (Arrows) (**Right**).

After examining the cardiac vasculature, coronary artery perfusion and percutaneous coronary intervention were performed to place stents if needed. Coronary angiography revealed that collateral circulation was formed on the pLAD through the right coronary artery due to chronic coronary occlusion (Figure 4). Based on these findings, the consulted cardiologist stated that the patient probably had good coronary flow at rest but developed cardiac ischemia during surgery due to severe hypotension that was induced by the anesthetics in a hypovolemic state. Because the coronary blood flow was maintained, no additional procedures were performed for coronary arteries. Cardiac parameter (troponin T) obtained before anesthesia and after waking from anesthesia was within a normal range (0.016 ng/mL before anesthesia, 0.016 ng/mL after anesthesia). Following the anesthesia attempt, the patient was hydrated with approximately 1.5 L of 0.9% normal saline daily for 5 days and was administered low molecular weight heparin (6000IU). At the second general anesthesia attempt, anesthesiologist used etomidate (0.2 mg/kg) for induction, as it has a lower impact on BP, and anesthesiologist used sevoflurane for maintenance. Both PVI and SVV indicated normovolemia before and after anesthesia, and a normal BP was maintained throughout the surgery.

## 3. Discussion

In this case, the patient had an undiagnosed coronary artery near-total occlusion and suspected dehydration. Upon administering propofol and sevoflurane for general anesthesia, severe hypotension occurred and, consequently, anesthesia was discontinued. Although the pre-anesthesia PVI (indicative of body fluid volume) indicated hypovolemia, the patient did not appear visibly ill. Therefore, the extreme changes in BP levels after anesthesia could not have been predicted.

Based on the pre-anesthesia EKG (Figure 1) of the patient, septal infarction was suspected on V2, but the ST segment on Lead II was unremarkable. The patient had near-total occlusion on the pLAD that had not been diagnosed prior to the anesthesia, but he was able to maintain normal BP at rest owing to collateral circulation.

However, administration of anesthetics (propofol and sevoflurane) in a hypovolemic state led to marked ST depression on EKG [7], and cardiac ischemia led to impaired cardiac functions, and ultimately, extreme hypotension. When a second general anesthesia was attempted after correcting hypovolemia, vital signs were relatively stable throughout the anesthesia, in contrast to the previous attempt.

Generally, anesthetics suppress cardiac functions and cause vasodilation, which in turn reduces venous return to the heart [8,9]. This mechanism can be more pronounced in a hypovolemic state. General anesthesia in a hypovolemic state not only leads to severe hypotension during anesthesia, but it can also result in insufficient oxygen supply, potentially leading to lactic acidemia and increased mortality post-anesthesia [10]. Hence, adequate hydration is crucial both before and during anesthesia [11].

Propofol was used for the first induction in the current case. Although propofol is globally used as an intravenous anesthetic for general anesthesia, it can lower BP and cardiac contractility and occasionally induce arrhythmias [1,12]. The patient in our case also showed ischemic changes on EKG and reduced BP after propofol administration. Even after administering sevoflurane for maintenance, BP remained unstable, and ischemic changes on the EKG persisted. Volatile anesthetics, such as sevoflurane, are known for their preconditioning effect, protecting the heart from harmful impacts of ischemia or hypoperfusion [13]. The present case suggests that the cardiac protection effect of anesthetics is meaningful only in normovolemic states. If there is collateral circulation formed for more than 25% of normal coronary arteries at rest, occlusion does not impact myocardial viability [14]. Furthermore, infarct size may decrease with collateral arteries, and even upon the onset of an infarction, collateral circulation may reduce complications or mortality [15,16]. However, it is unclear if collateral circulation can maintain adequate cardiac blood supply during exercise or stress [17,18]. Even in the case of this patient, echocardiography at rest, a few hours after anesthesia, showed a normal right ventricle and an LVEF of 45–50%, indicating no remarkable abnormalities. This case highlights the importance of thoroughly investigating risk factors for cardiac complications during anesthesia, including old age, renal dysfunction, and DM management with insulin [19,20]. If such risk factors are identified, it may be necessary to refer the patient for a cardiologist consultation, which should include both resting echocardiography and stress echocardiography using dobutamine until initiating NPO status for surgery [19,20,21]. This can help in assessing the precise extent of cardiac function impairment during general anesthesia [21,22]. For instance, a study reported surgery cessation due to the onset of myocardial infarction in a patient undergoing a surgery with potential for major bleeding, such as hepatectomy [23]; therefore, a cardiac assessment, such as stress echocardiography, should be performed preoperatively in patients scheduled for high-risk surgery, who have not been diagnosed with a cardiac disease but do exhibit the risk factors. In addition, another study reported an unexpected cardiac arrest after induction of general anesthesia in a young patient without a remarkable medical history [24]. Hence, it is advisable to prepare vasopressors in advance to facilitate their prompt administration in the event of unexpected severe hypotension.

One point for further discussion in our case is regarding whether the reduction in BP would not have occurred if etomidate or ketamine [25,26], which are associated with less dramatic changes in vital signs, was used instead of propofol in a dehydrated patient with CAD. However, since the SBP again dropped to the 60s even after using sevoflurane for maintenance of anesthesia, we suspect that hypovolemic state and coronary artery occlusion would have had a greater impact on the hemodynamic instability, as opposed to the type of anesthetic used. Moreover, one adverse effect of ketamine is that cardiac dysfunction can be exacerbated in patients with a pre-existing cardiac condition [27]. Furthermore, while etomidate has lower effects on BP changes in the presence of impaired cardiac functions and hypovolemia, it may increase long-term mortality due to adrenocortical suppression; thus, propofol is generally chosen if BP is within normal ranges before anesthesia [28].

However, as the decision in our second attempt of general anesthesia, etomidate or ketamine may be considered for patients who are anticipated to develop hemodynamic instability during anesthesia due to heavy bleeding or dehydration before anesthesia [25,29]. A limitation of our study is that only lead II is monitored on EKG during anesthesia, and it cannot provide accurate measurements of changes in EKG waveforms, which hinders an accurate determination of ischemic changes. However, the patient in our case showed marked ST depression that had not been observed before anesthesia (Figure 1) and CT heart angio taken after anesthesia revealed near total occlusion of the left descending artery. Based on these findings, we concluded that the anesthetic led to transient cardiac ischemia and dysfunction, which in turn caused a drop in BP.

## 4. Conclusions

We found that for a patient with coronary artery occlusion who maintains normal BP at rest owing to collateral circulation, administration of anesthetics in a hypovolemic state could lead to severe hypotension that may potentially cause cardiac arrest. Thus, for older patients with a high likelihood of CAD, more thorough cardiac examinations, such as stress echocardiography, should be performed in addition to the routine pre-anesthesia tests for an accurate assessment of cardiac functions. Furthermore, the compensatory cardiovascular system in response to a decrease in BP due to anesthetics may be impaired in older patients, and timely response to a drastic drop in BP after induction of anesthesia may be hindered. Thus, it is important to thoroughly examine patients for hypovolemia, provide adequate hydration, and prepare vasopressors before induction of anesthesia to ensure a timely response to BP reduction after the administration of anesthetics.

## Data Availability

Data are contained within the article.
